# IL-6 Affects Liver Metabolic Abnormalities Caused by Silicon Exposure by Regulating the PKC/YY1 Signaling Pathway

**DOI:** 10.3390/genes16040456

**Published:** 2025-04-16

**Authors:** Hui Zhao, Huihui Tao, Jian Gao, Jingjing Wang, Guangliang Hui, Ye Zhu, Jialin Wang, Xuansheng Ding, Yong Dai

**Affiliations:** 1 Department of Pharmacology, School of Medicine, Anhui University of Science and Technology, Huainan 232001, China; zh_2002137@126.com (H.Z.); uniquethh@aust.edu.cn (H.T.); gaojian@aust.edu.cn (J.G.); wang12320250103@163.com (J.W.); hgl48749055751@163.com (G.H.); zhye12366@163.com (Y.Z.); wjl1981214976@163.com (J.W.); 2 Key Laboratory of Industrial Dust Deep Reduction and Occupational Health and Safety of Anhui Higher Education Institutes, Huainan 232001, China; 3 Anhui Province Engineering Laboratory of Occupational Health and Safety, Huainan 232001, China; 4 Department of Pharmacology, China Pharmaceutical University, Nanjing 211198, China; 5 Joint Research Center for Occupational Medicine and Health of IHM, Anhui University of Science and Technology, Huainan 232001, China

**Keywords:** coal dust exposure, dyslipidemia, lipid metabolism, FMO3 (flavin monooxygenase 3), IL-6 (interleukin-6)

## Abstract

Background: This study aims to investigate the impact of coal dust (silicon dioxide) exposure on dyslipidemia and its underlying mechanisms, with a focus on the association between coal dust exposure and hepatic metabolic disorders. Methods: Clinical data were collected from 5433 coal mine workers to compare the incidence of dyslipidemia between the dust-exposed group and the non-exposed group. A mouse model of silicon dioxide exposure was established to observe hepatic fat accumulation and pathological changes. Liver tissue sequencing was performed to screen for key differential genes. In vitro cell experiments were utilized to identify the molecular mechanisms underlying hepatocyte metabolic abnormalities induced by silicon dioxide exposure. Results: Clinical data revealed that 69.2% of miners in the dust-exposed group developed dyslipidemia, which was higher than the 30.7% in the non-exposed group. Animal data showed that silicon dioxide exposure led to hepatic fat deposition and pathological damage, with the degree of injury positively correlated with exposure time. Liver sequencing identified a significant upregulation of the FMO3 (flavin monooxygenase 3) gene in mouse liver tissue following silicon dioxide exposure, accompanied by enhanced inflammatory responses. Mechanistic studies demonstrated that silicon dioxide activates Kupffer cells to secrete IL-6 (interleukin-6), which induces high expression of FMO3 in hepatocytes through the PKC/YY1 signaling pathway, thereby disrupting lipid metabolism. Conclusions: Silicon dioxide exposure can promote the upregulation of FMO3 expression in hepatocytes by activating Kupffer cells to release IL-6 via the PKC/YY1 pathway, ultimately leading to lipid metabolic disorders and dyslipidemia

## 1. Introduction

Cardiovascular diseases, as serious and highly prevalent conditions, have dyslipidemia as a key cause of their pathogenesis [1]. The formation of dyslipidemia is influenced by various factors, including genetics, environment, and an unhealthy lifestyle. Recent studies report that people, such as coal miners, who are under long-term exposure to coal (silica) dust, have significantly higher incidence rates of coronary heart disease, cerebrovascular diseases, atherosclerosis, and hypertension than the general population [2,3]. This suggests that exposure to coal (silica) dust may be a key factor in inducing dyslipidemia, and further research is warranted.

Previous studies have reported that after the inhalation of coal (silica) dust particles into the lungs, they are engulfed by alveolar macrophages, leading to cell activation and the release of inflammatory factors, such as interleukin-6 (IL-6) and tumor necrosis factor-α (TNF-α), which can trigger local inflammatory responses [4]. Concurrently, inflammation leads to endothelial dysfunction, affecting the vasomotion and permeability of blood vessels, as well as coagulation and fibrinolysis processes, thereby impacting the metabolism and distribution of blood lipids [5]. Consistently, pulmonary inflammatory responses can activate the immune system, leading to the occurrence of autoimmune reactions [6].

The activation of autoimmune reactions can attack normal tissues and cells, including those involved in lipid metabolism such as liver and immune cells, thereby indirectly affecting blood lipid levels [7]. The latest research has found that particulate crystalline silica (CS) serves as the primary pathogenic factor in coal (silica) dust [8]. Once CS enters the lungs through the primary respiratory route, a portion of CS is ingested by pulmonary macrophages, while the rest of the CS can rapidly enter the bloodstream through circulation [9]. Typically, the concentration of silica particles in the serum of normal adults ranges from 100 to 500 μg/L, whereas the level of silica particles in the serum of populations exposed to coal (silica) dust is significantly higher than that of normal adults, such that the average level of silica particles in the serum of patients with silicosis caused by exposure to coal (silica) dust can reach up to 4230 μg/L [10,11].

The liver, being one of the main organs in the circulatory system, can also accumulate silica particles. Consistent with this, studies have reported that silica particles enter the liver through the bloodstream and are phagocytosed by Kupffer cells. This activates inflammatory responses and the production of oxidative stress, thereby damaging hepatocytes [12].

Additionally, liver damage caused by silica nanoparticles (SiNPs) is also related to their composition and size [13]. Research indicates that SiNPs are capable of inducing inflammation in the liver, lipid accumulation, and fibrosis, along with elevated levels of ALT, AST, total cholesterol (TC), and triglycerides (TG) [14].In summary, silica particles may be a key pathogenic factor leading to liver metabolic abnormalities due to exposure to coal (silica) dust, potentially affecting liver function by inducing pulmonary immune inflammation or by directly targeting liver-related cells, yet the specific mechanisms of action remain unclear.

This study aims to analyze clinical lipid data from populations exposed to coal (silica) dust to identify risk factors causing dyslipidemia. An animal model of liver metabolic disorders caused by silica exposure is constructed, and key genes affecting liver metabolism are identified using tissue sequencing and in vitro cell models. The mechanisms by which they act on targets both in vitro and in vivo are studied, with the ultimate goal of providing new strategies for the prevention and treatment of cardiovascular diseases caused by exposure to coal (silica) dust.

## 2. Materials and Methods

### 2.1. Collection of Clinical Data and Blood Samples

This study collected health examination data and clinical sample information from employees in three coal mining areas in Huainan City between 2020 and 2023. The inclusion criteria for the study population were as follows: (1) Non-exposed individuals: administrative staff of the company with no history of coal dust exposure. (2) Coal dust-exposed individuals: frontline mining workers of the company with a recorded history of coal dust exposure. A total of 5657 employees were included. Based on the exclusion criteria, which included (1) incomplete health examination data and (2) presence of diseases affecting blood lipids, including hypothyroidism, diabetes, and kidney diseases, 5357 employees met the requirements [15]. The employees included miners, logistical staff (water and electricity, canteen, maintenance), and administrative staff. The population was divided into two groups based on job types: the dust-exposed group (miners) and the non-dust-exposed group (logistical and administrative staff). In terms of clinical information collection, basic data were gathered from 5357 employees, including gender, age, BMI, BP, occupation, lifestyle, dietary habits, and medical history. Within the health examination data, participants were further categorized into two groups based on lipid profiles: the normal lipid group and the abnormal lipid group. The diagnostic criteria for abnormal blood lipids were triglyceride (TG) levels greater than 1.7 mmol/L or cholesterol levels (TC) greater than 5.2 mmol/L; meeting any of these conditions was considered abnormal blood lipids. Following this criterion, the 5357 employees were classified into the normal lipid group (*n* = 4433) and the abnormal lipid group (*n* = 924).

In the collection of blood samples, we selected blood samples from 20 non-dust-exposed and 20 dust-exposed employees in the normal blood lipid group, as well as from 20 non-dust-exposed and 20 dust-exposed employees in the dyslipidemia group for analysis. Additionally, all participants underwent physical examinations and laboratory tests. This study has been approved by the Biomedical Research Ethics Committee of Anhui University of Science and Technology (Ethics No.: 2021032), and all participants signed informed consent forms before the study began. Additionally, all experiments were conducted in accordance with the Declaration of Helsinki and relevant national and international ethical guidelines and regulations.

### 2.2. Experimental Animals and Materials

Eleven male C57BL/6 mice were procured from Hefei Qingyuan Biotechnology Co., Ltd., (Hefei, China), aged 8–10 weeks with initial body weights of 20–22 g. All animals were housed in SPF-grade barrier facilities at Anhui University of Science and Technology with ad libitum access to food/water under 12 h/12 h light/dark cycles prior to experimentation. Environmental parameters were maintained at 22 ± 1 °C with 50 ± 10% relative humidity. Mice were group-housed (3–4/cage) in Tecniplast GM500 individually ventilated cages. Autoclaved corncob bedding (Biofresh, Dalian, China) was replaced daily with systematic health monitoring. Crystalline silica particles (Sigma #S5631, >99% purity, St. Louis, MO, USA) with size distribution of 0.5–10.0 μm (80% within 1.0–5.0 μm) were employed in this model. Particles underwent dry-heat sterilization (100 °C, 1 h) followed by suspension in sterile PBS to 50 mg/mL concentration. The silica suspension was ultrasonicated for 10 min to ensure homogeneous dispersion prior to administration.

### 2.3. Silica Exposure Mouse Model Inducing Liver Injury Construction and Analysis

In the CS-induced hepatotoxicity model, mice were randomly divided into three groups using complete randomization: PBS control (*n* = 3), 30-day CS exposure (CS-1M, *n* = 4), and 60-day CS exposure (CS-2M, *n* = 4). Anesthesia was induced with 3% isoflurane (RWD #R510-22-10) in a sealed chamber, maintained at 1.5% for 5–10 s until respiratory stabilization. Mice were gently restrained for nasal instillation of 50 μL PBS/CS suspension administered in 4–5 aliquots using a micropipette with a precision dosing technique [16]. Post-instillation thoracic massage was performed until complete recovery. The procedure induced transient stress responses without causing tissue damage, eliminating analgesic requirements. Furthermore, the progressive nature of silica-induced chronic hepatopathy represents a non-acute pathological process requiring no analgesia [17]. Post-modeling monitoring included daily weight/behavior assessments, with predefined euthanasia criteria (≥20% weight loss, severe dyspnea, or mobility impairment); no animals met these endpoints [18].

On the 30th and 60th days of the experiment, mice from the CS-1M and CS-2M groups were euthanized by cervical dislocation after being deeply anesthetized with 1.5% isoflurane inhalation (RWD, #R510-22-10), which is a euthanasia method for small rodents recognized by the AVMA Guidelines 2020. Lung, liver, and blood samples were collected for subsequent analysis. All animal experiments were approved by the Experimental Animal Ethics Committee of Anhui University of Science and Technology (Approval No. GZ2022-020). All methods were performed in accordance with the ARRIVE guidelines (https://arriveguidelines.org (accessed on 25 May 2024)) and relevant national/international regulations for the care and use of laboratory animals. Mice were housed under SPF conditions with ad libitum access to food and water. Procedures involving anesthesia (isoflurane) and euthanasia (cervical dislocation under deep anesthesia) strictly followed the AVMA Guidelines 2020 to minimize animal suffering.

### 2.4. Hematoxylin and Eosin (HE) Staining and Oil Red O Staining

Mouse liver and lung tissue samples were collected, fixed with 4% paraformaldehyde, then dehydrated and paraffin-embedded. Samples were stained with hematoxylin and eosin (HE) staining solution (Beyotime, #C0105M, Shanghai, China) for 5 min, respectively, then rinsed with running water for 5 min. The slides were mounted and observed and photographed under a light microscope (OLYMPUS), where the cell nuclei appear blue and the cytoplasm appears red. For Oil Red O staining of liver tissue, frozen sections of liver tissue were prepared using a cryostat (Leica, #CM1950, Wetzlar, Germany) with section thicknesses of 5–10 µm then stained with Oil Red O solution (solarbio, #G1261, Beijing, China) for 8 to 10 min (slides were covered to avoid light exposure during this process). After staining, they were rinsed with running water for 5 min and then dipped in 60% isopropanol for 3–5 s for background differentiation. The slides were mounted and observed and photographed under a light microscope (OLYMPUS, Tokyo, Japan); lipids appeared red. The results of HE and Oil Red O staining were analyzed using ImageJ software (V1.5.2). Specifically, during the HE analysis, detailed statistics on lung nodules and their corresponding areas were gathered. The relative area of the lung nodules was then derived by dividing the total nodule count by the cumulative nodule area. As for the Oil Red O staining analysis, the proportion of ORO-positive staining regions to the overall tissue area was computed, thereby quantifying the relative expression area of ORO.

### 2.5. Assessment and Analysis of Lipid Levels in Mice

After the feeding period, mice fasted for 12 h with free access to water to standardize experimental conditions. Subsequently, mice were anesthetized with isoflurane, followed by enucleation and peripheral blood collection. The collected blood samples were centrifuged at 4 °C to separate the serum. Using a biochemical analyzer (BS-240, Mindray Corporation, Shenzhen, China), we measured multiple biochemical indices in the serum, including levels of total cholesterol (TC), triglycerides (TG), low-density lipoprotein cholesterol (LDL-C), high-density lipoprotein cholesterol (HDL-C), alanine aminotransferase (ALT), and aspartate aminotransferase (AST). The assay kits were purchased from Mindray Biomedical Electronics Co., Ltd., (Shenzhen, China), with the following batch numbers: total cholesterol (# 141622013), triglycerides (#141721003), alanine aminotransferase (#140121005), aspartate aminotransferase (#140222012), high-density lipoprotein (#142122002), and low-density lipoprotein (#142022003).

### 2.6. Mouse Liver Tissue RNA Sequencing and Analysis

Mouse liver tissue samples were collected and isolated, and total RNA was purified using the TRIzol reagent (Thermo Fisher, #15596018) according to the manufacturer’s protocol. Post-purification, the total RNA samples are quality-controlled for quantity and purity using the NanoDrop ND-1000 (NanoDrop, Wilmington, DE, USA), and the integrity of the RNA was assessed with the Bioanalyzer 2100 (Agilent, Santa Clara, CA, USA). Samples met the requirements for subsequent experiments only when the RNA concentration exceeded 50 ng/μL, the RIN value was greater than 7.0, and the total RNA amount was over 1 μg. Oligo(dT) magnetic beads (Dynabeads Oligo (dT), #25-61005, Thermo Fisher, Waltham, MA, USA) were utilized to specifically capture PolyA-tailed mRNA through two rounds of purification. The captured mRNA was fragmented under high-temperature conditions using the Magnesium RNA Fragmentation Module (NEBNext, #E6150S, Ipswich, MA, USA), with a treatment at 94 °C for 5–7 min. The fragmented RNA was reverse-transcribed into cDNA by the action of Invitrogen SuperScript II Reverse Transcriptase (#1896649, Carlsbad, CA, USA). Subsequently, second-strand cDNA synthesis was carried out using *E. coli* DNA polymerase I (NEB, #m0209, Ipswich, MA, USA) and RNase H (NEB, #m0297, Ipswich, MA, USA), incorporating dUTP Solution (Thermo Fisher, #R0133, CA, USA) into the second strand to create blunt ends. After end-repair, an A base was added to facilitate ligation with adapters that have a T base at their 3′ end. To further process the cDNA, we digested uracil in the double strands with UDG enzyme (NEB, #m0280, Ipswich, MA, USA), followed by PCR amplification: initial denaturation at 95 °C for 3 min, then 8 cycles of 98 °C denaturation for 15 s, annealing at 60 °C for 15 s, and extension at 72 °C for 30 s, with a final extension at 72 °C for 5 min to form a size-specific library with fragments of 300 bp ± 50 bp. Double-end sequencing was performed using the Illumina NovaSeq 6000 (LC Bio Technology Co., Ltd., Hangzhou, China) with a PE150 pattern. In bioinformatics analysis, all volcano plots, bar charts, and heatmaps are generated using R software (R-project) on the OmicStudio platform. Refer to Supplementary Table S1 for the original sequencing data.

### 2.7. Analysis of Immunofluorescent Staining

After deparaffinization and antigen retrieval of 5 μm mouse liver tissue paraffin sections, 10% BSA was used for blocking to reduce nonspecific binding. Sections were incubated at room temperature at 37 °C for 30 min to complete the blocking. Subsequently, we applied appropriately diluted primary antibodies in a humidified chamber: FMO3 (Abcam, #ab126711, 1:150, Cambridge, UK), F4/80 (Abclonal, #A1256, 1:200, Woburn, MA, USA), and IL-6 (Abclonal, #A0286, 1:200). Samples were then incubated overnight at 4 °C to ensure adequate binding. After overnight incubation, they were washed twice with PBS, and then the corresponding fluorescently labeled secondary antibodies were added: FITC-labeled Goat Anti-Rabbit IgG (1:500, #GB22303, Servicebio, Hangzhou, China) and Cy5-conjugated Goat Anti-Rabbit IgG (1:500, #GB27303, Servicebio). Incubation at room temperature was continued for 1 h to form the immune complexes. Then, the cell nuclei were stained with DAPI (CST, #4083S) for 5 min. After the samples were mounted with 50% glycerol, images of the samples were observed and captured using an upright fluorescence microscope (Nikon Eclipse C1, Tokyo, Japan). The intensity of the fluorescence signals was analyzed using the ImageJ software (V1.5.2), and the experimental results were quantified.

### 2.8. RT-qPCR

For mouse liver tissue, 20–40 mg of freshly cut tissue was taken and ground thoroughly in liquid nitrogen for 5 min. Then, 1 ml of Trizol reagent (Thermo Fisher Scientific, Waltham, MA, USA) was added and lysed on ice for 10 min. For cell samples, 1 mL of Trizol reagent was added to 1 × 10^5^ to 1 × 10^6^ cells and lysed on ice for 5 min. Subsequently, 300 μL of chloroform were added in a 10:3 ratio, mixed well, let stand for 10–15 min, and then centrifuged at 12,000× *g* at 4 °C for 20 min. Approximately 200–400 μL of the supernatant were carefully aspirated from the intermediate layer where the white RNA is located. After precipitating with isopropanol in a 1:1 ratio, the RNA precipitate was washed twice with 1 ml of pre-cooled ethanol (75%). Finally, the RNA was dissolved in 20–40 μL of DEPC water, and the concentration and quality of the RNA were measured using a spectrophotometer. An OD260/OD280 ratio between 1.8–2.0 and a concentration of 200–500 ng/μL indicate that the RNA quality meets the requirements. Following this, reverse transcription experiments were performed using the PrimeScript™ RT reagent Kit (Takara, #RR037A, Dalian, China). The reverse-transcribed cDNA was amplified using the TB Green^®^ Premix Ex Taq™ II kit (Takara, #RR820A, Dalian, China) on the QuantStudio 3 Real-Time PCR System (Thermofisher). All primers were designed using the PrimerBank database (https://pga.mgh.harvard.edu/primerbank/ (accessed on 12 January 2024) for online design, with a complete list of primers provided in Supplementary Table S2.

### 2.9. Cell Culture and Treatment

Human liver normal cells THLE-3 were purchased from the ATCC cell repository (#CRL-11233, Manassas, VA, USA), and human liver Kupffer cells (#SNP-H065) were purchased from Sun Biotech Company. THLE-3 cells were cultured in DMEM high glucose medium (Hyclone) + 10% fetal bovine serum (Gibco) + 1% double antibody (Hyclone), at 37 °C, 5% CO_2_ incubator (Panasonic). Human liver Kupffer cells were cultured using primary cell complete medium (SUNNCELL, #SNPM-H065, Shanghai, China). After treating Kupffer cells with CS (50 µg/cm^2^) for 48 h, the supernatant was collected and centrifuged at 4 °C, 1000 rpm, and the supernatant was taken and then prepared as a conditioned medium (CM) with a DMEM/supernatant ratio of 1:1 before use. Recombinant human IL6 protein was purchased from MCE Company (#HY-P7044, Monmouth Junction, NJ, USA) and diluted with PBS to a storage solution of 5 µg/mL before use. The IL6R inhibitor (tocilizumab, Roche, Basel, Switzerland) was purchased from MCE Company (#HY-P9917) and diluted with PBS to a storage solution of 5 mg/mL before use. The working concentrations of recombinant human IL6 protein and tocilizumab for treating THLE-3 cells were 5 ng/mL and 10 ng/mL, respectively.

### 2.10. Western Blot Experiment

The treated THLE-3 and Kupffer cells were collected, and RIPA lysis buffer (Beyotime, #P0013C, Haimen, China) was added to lyse cells on ice for 10 min. The cells were scraped with a cell scraper and centrifuged at 4 °C, 14,000 rpm, for 25 min, and the supernatant was then taken. The protein concentration was measured using the BCA Protein Assay Kit (Thermo Fisher Scientific, #A55864), and a total of 20–40 μg of protein was extracted. After separation by 10% SDS-PAGE, it was then transferred to a 0.2 μm PVDF membrane (Immobilon, #ISEQ00010, Burlington, MA, USA). The membrane was blocked with 5% non-fat dry milk at room temperature, then washed with TBST. The membrane was incubated with specific primary antibodies overnight at 4 °C, then washed with TBST. The membrane was then incubated with secondary antibodies in 5% non-fat dry milk, then incubated with ECL (Millipore, #WBKLS0100, Burlington, MA, USA) for 30 s. An imaging system (Amersham™ ImageQuant™ 800, #29399481, Little Chalfont, UK) was used to visualize the protein bands. The primary antibodies used include the following: FMO3 (Abcam, #ab126711, 1:1000), IL-6 (Abclonal, #A0286, 1:1000), PBX2 (PTG, #22321-1-AP, 1:500), YY1 (Abclonal, #A19569, 1:1000), USF1 (PTG, #22327-1-AP, 1:500, Rosemont, IL, USA), Phospho-Akt-T308 (Abclonal, #AP1259, 1:500), Phospho-PKC α-T638 (Abclonal, #AP137, 1:2000), and β-Actin (ABclonal, #AC026, 1:10,000), and the secondary antibody used was HRP-conjugated. All strips were displayed after being cut, and the band grayscale values were analyzed using ImageJ software (V1.5.2). Specifically, western blot (WB) results were converted into 8-bit grayscale images, using β-Actin as an internal reference protein. The ratio of the grayscale values between the target protein band and the corresponding internal reference band was calculated. Data normalization was performed using the means ± SEM from three independent experiments.

### 2.11. ELISA Detection of Inflammatory Cytokines in Human and Mouse Serum

Enzyme-linked immunosorbent assay (ELISA) kits from Abcam were used to detect the expression levels of inflammatory cytokines (IL-1α, IL-1β, IL-6, TNF-α, and TGF-β) in the serum of individuals exposed and not exposed to dust in both normal and abnormal lipid groups. Additionally, the expression levels of inflammatory cytokines (IL-1β, IL-6, TNF-α, and TGF-β) were measured in the serum and liver tissue of mice in the PBS, CS-1M, and CS-2M groups. The experimental procedures were strictly followed according to the kit instructions, including the generation of standard curves. Based on these standard curves, the concentrations of various inflammatory cytokines in the samples were calculated. The human ELISA kits used were as follows: IL-1α (abcam, #ab100560), IL-1β (abcam, #ab214025), IL-6 (abcam, #ab178013), TNF-α (abcam, #ab46087), and TGF-β (abcam, #ab100647). The mouse ELISA kits used were: IL-1β (abcam, #ab197742), IL-6 (abcam, #ab100713), TNF-α (abcam, #ab208348), and TGF-β (abcam, #ab119557).

### 2.12. GSEA and TRRUST Databases

The Gene Set Enrichment Analysis (GSEA) database, accessible at https://www.gsea-msigdb.org/gsea/index.jsp, accessed on 14 March 2024, is a tool for analyzing whole-genome expression profile data. The terms “metabolism” or “inflammation” were entered in the keywords search bar on the site. The search filters were set to the following parameters: collection = all collections, source species = Homo sapiens, and contributor = all contributors. This retrieved 342 gene sets. They were downloaded in GMT format, then imported into EXCEL. After duplicate genes were removed, 2194 metabolism-related genes and 4867 inflammation-related genes were identified, listed in Supplementary Table S3. TRRUST (Transcriptional Regulatory Relationships Unraveled by Sentence—based Text mining), available at http://www.grnpedia.org/trrust/ (accessed on 14 March 2024), is a manually-curated database of transcriptional regulatory networks. It includes transcription factors’ target genes and the regulatory relationships between transcription factors. On the TRRUST site, FMO3 was entered in the “Search a gene in TRRUST database” section, setting the species to human. This yielded the key transcription factors regulating FMO3.

### 2.13. Statistical Analysis

All statistical analyses were conducted using GraphPad Prism 8.0.2 software. Data are presented as mean ± standard error of the mean (SEM). Statistical analysis was conducted using *p*-values determined by independent sample *t*-tests, one-way analysis of variance (one-way ANOVA), and two-way analysis of variance (two-way ANOVA), A *p*-value of ≤0.05 was considered statistically significant (ns, not significant; * *p* ≤ 0.05; ** *p* ≤ 0.01; *** *p* ≤ 0.001).

## 3. Results

### 3.1. Characterization of Dust-Exposed Workers

**Figure 1:**
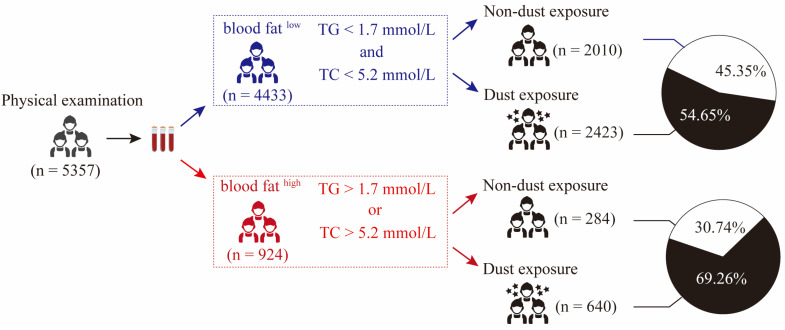
Analysis of clinical factors influencing abnormal blood lipids. Analysis of the proportion of dust exposure and related factors in individuals with abnormal blood lipids.

Clinical studies have shown that individuals exposed to coal dust often exhibit dyslipidemia [19]. This finding aligns with the results from our previous research [15], where we collected and screened the medical examination data of 5433 workers from three coal mining areas in Huainan City (Supplementary Figure S1) to investigate the correlation between coal dust exposure and dyslipidemia. Based on serum levels of triglyceride (TG) and total cholesterol (TC), the subjects were divided into two groups: the normal lipid group (*n* = 4433, represented in blue) and the dyslipidemia group (*n* = 924, represented in red). Considering that the workforce consists of miners and support personnel, we further subdivided the population into dust storm exposure and non-dust storm exposure groups based on their job responsibilities. It is noteworthy that among the normal lipid group, only 54.65% were exposed to dust storms, whereas this proportion significantly increased to 69.26% in the dyslipidemia group (Figure 1). Meanwhile, the proportion of the dust storm-exposed population in the dyslipidemia group (69.26%) was significantly higher than that of the non-dust storm-exposed population (30.73%) (Figure 1). Furthermore, in our previous study [15], univariate analysis revealed that dust exposure, age, BMI, blood pressure, and smoking were statistically significant risk factors for dyslipidemia (*p* < 0.05). However, analysis of three multivariable models adjusted for different confounding factors (dust exposure, age, BMI, blood pressure, and smoking) found that coal dust exposure was a key factor contributing to the increased risk of dyslipidemia among miners. In summary, exposure to dust storms may be a potential risk factor for the development of dyslipidemia.

### 3.2. Silicon Exposure-Induced Dyslipidemia and Liver Dysfunction in Mice

**Figure 2:**
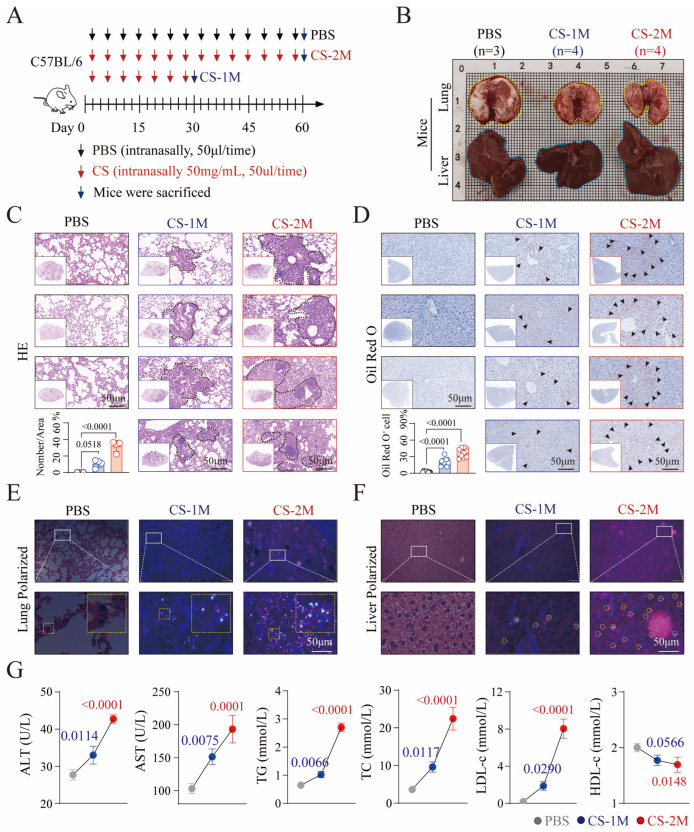
Histopathological changes and liver function analysis in silica-exposed mice. (**A**) Construction and analysis of a mouse model of liver dysfunction caused by silica exposure. After inhalation anesthesia of the mice, PBS (50 μL per administration) and CS (50 mg/mL, 50 μL per administration) were administered via gavage every three days and stopped after 30 and 60 days, respectively. After inhalation anesthesia of the mice, PBS (50 μL per administration) and CS (50 mg/mL, 50 μL per administration) were administered via nasal drip every three days and stopped after 30 and 60 days, respectively. (**B**) The appearance of the lungs and livers of three groups of mice: PBS (*n* = 3), CS-1M (*n* = 4), and CS-2M (*n* = 4), with the yellow and blue dashed lines indicating the areas of lung and liver tissues, respectively. (**C**) HE staining and analysis of lung tissue from three groups of mice. The inset shows a high magnification image of the framed area, with the black dashed line indicating the pulmonary nodule. The bar graph at the bottom left shows the relative expression of nodule count/nodule area in lung tissue, with each point representing a mouse. (**D**) Oil Red O staining and analysis of liver tissue from three groups of mice. The inset shows a high magnification image of the framed area, with black arrows indicating areas of fat accumulation. The bar graph at the bottom left shows the statistical chart of the relative expression of fat accumulation in liver tissue, with three fields of view selected for analysis per mouse and each point representing a field of view. (**E**) Polarized light detection and analysis of lung tissue from three groups of mice. The bottom side shows a high magnification image of the area enclosed by the white solid line, with the inset displaying a high magnification image of the area enclosed by the yellow dashed line and the white spots indicating the deposition and aggregation of silica particles. (**F**) Polarized light detection and analysis of liver tissue from three groups of mice. The base shows a high magnification image of the area enclosed by the white solid line, with the yellow dashed circles indicating the deposition and aggregation of silica particles. (**G**) Detection and analysis of blood lipids (TC, TG, LDL-c, and HDL-c) and liver function indicators (AST and ALT) in three groups of mice. Scale bar, 50 micrometers (**C**–**F**), 1000 micrometers (**B**). Data are presented as mean ± SEM, and *p*-values and significance are determined by one-Way ANOVA (**C**,**D**,**G**).

There is a current lack of literature accurately reporting the effects of coal dust exposure on the blood lipids and liver function in mice. However, studies have found that after 28 days of inhaling CS, the main pathogenic component in coal dust, rats show a significant aggregation of silicon particles in the lungs accompanied by pathological changes, while only a small amount of silicon particles have been found in the liver [20]. Based on this, we used micrometer-level CS (50 mg/mL) and stimulated mice through nasal drop (once every 3 days, 50 μL each time) for 30 days (CS-1M) and 60 days (CS-2M), respectively, to observe changes in lung and liver tissues (Figure 2A). The results showed that as the duration of CS stimulation increased, the surface of the mouse lung tissue (indicated by yellow dashed lines) exhibited obvious nodules and a rough texture. Although the liver tissue volume increased (indicated by blue dashed lines), no obvious pathological changes were observed on the surface (Figure 2B).

The HE staining results further confirmed that compared to the PBS group, there was a substantial increase in the number of lung tissue nodules (indicated by black dashed lines) in the CS-1M and CS-2M groups of mice, and the number of nodules increased gradually with the extension of CS stimulation (Figure 2C). Similarly, Oil Red O staining of liver tissue showed that mice in the CS-1M group had slight fat accumulation and hepatic steatosis (Figure 2D, middle, black arrow), but mice in the CS-2M group (Figure 2D, right, black arrow) exhibited significant pathological changes in liver tissue. This suggests that liver tissue lesions may lag behind lung tissue lesions in time. This may be due to CS first attacking the lungs [8], and after entering the lungs, CS can break through the lung barrier, enter the bloodstream, and deposit in various tissues [21].

Consequently, we employed polarized light to examine the deposition and aggregation of silicon particles in the lung and liver tissues. The results showed that compared to the PBS group, there was a significant deposition and aggregation of silicon particles in the lung tissues of CS-1M and CS-2M mice, which increased progressively with the extension of CS stimulation (Figure 2E, white spots), consistent with previous pathological results of lung tissue (Figure 2C). Similarly, compared to the CS-1M group, there was a clear deposition and aggregation of silicon particles in the liver tissues of CS-2M mice (Figure 2F, yellow dashed line), which was consistent with the previous pathological changes in liver tissue (Figure 2D). This suggests that silicon particles may be a key factor in causing liver tissue lesions.

Given that liver tissue lesions can lead to lipid metabolism disorders and abnormal blood lipids [22], we measured the levels of blood lipids (TC, TG, LDL-c, and HDL-c) and liver function indicators (AST and ALT) in mice [1]. Compared with the PBS group, the levels of TC and TG in CS-1M and CS-2M mice showed a gradually increasing trend, which was more pronounced in the CS-2M group. At the same time, the level of LDL-c gradually increased, while the level of HDL-c significantly decreased (Figure 2G). Furthermore, the levels of AST and ALT were also abnormally high, further suggesting liver function impairment and lipid metabolism abnormalities caused by CS stimulation.

### 3.3. Key Genes Associated with the Occurrence of Hepatic Metabolic Dysfunction in Silicon-Exposed Mice

**Figure 3:**
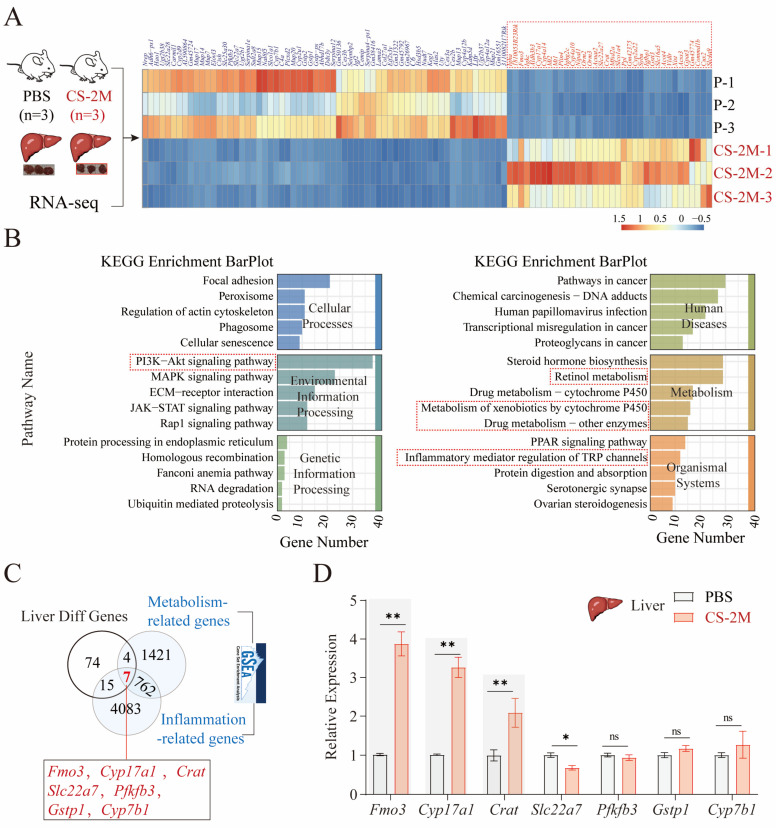
Screening of key genes affecting metabolism in liver tissue of mice exposed to silicon. (**A**) Sequencing and analysis of liver tissues from mice in the PBS (*n* = 3) and CS-2M (*n* = 3) groups, with the right-side heatmap illustrating the TOP 100 differentially expressed genes (|log2FC| > 1, *p* < 0.05); red indicates upregulated genes, and blue indicates downregulated genes. (**B**) Enrichment analysis of the KEGG pathways for the TOP 100 genes. (**C**) Intersection graph of genes related to “metabolism” and “inflammation” pathways in the GSEA database with the first 100 differentially expressed genes in liver tissue. (**D**) Bar chart of the expression of seven genes in liver tissue from mice in the PBS (*n* = 3) and CS-2M (*n* = 3) groups, with experiments conducted in triplicate. Data are presented as mean ± SEM, with *p*-values and significance determined by two-way ANOVA. ns, not significant; * *p* ≤ 0.05; ** *p* ≤ 0.01.

To analyze the key genes affecting hepatic metabolic functions in silicon-exposed mice, we examined the differentially expressed genes (|log2FC| > 1, *p* < 0.05) in liver tissues from the PBS (*n* = 3) and CS-2M (*n* = 3) groups. The results indicated that the Fmo3 gene, Cyp family genes (Cyp17a1, Cyp4a14, and Cyp4a10), and Slc family genes (*Slc22a27*, *Slc16a5*, and *Slc4a9*) were significantly upregulated (Figure 3A). FMO3 and the CYP family play a pivotal role in liver metabolism, detoxification, and the maintenance of overall physiological balance [23]. SLC family genes, acting as transport proteins, can affect the liver’s ability to transport substances, thereby influencing liver function [24]. KEGG pathway enrichment analysis revealed that lipid metabolism, inflammation-related pathways, and the PI3K-AKT signaling are key pathways for gene enrichment (Figure 3B). This suggests that lipid metabolism and inflammatory responses may be key pathways for liver dysfunction in silicon-exposed mice.

Consistent with this, studies have reported that excessive accumulation of adipose tissue can induce a state of chronic low-grade inflammation [25]. Inflammation can increase cellular uptake and accumulation of lipids, leading to an excessive buildup of lipids within cells [26]. Additionally, it has been reported that the PI3K/AKT pathway can promote inflammation triggered by oxidative stress, thereby exacerbating lipopolysaccharide-induced sepsis and liver failure [27]. This suggests that lipid metabolism and inflammatory responses may be key pathways for liver dysfunction in silicon-exposed mice. Accordingly, we searched the GSEA database for genes related to “Metabolism” and “Inflammation” and intersected them with differentially expressed genes in liver tissue. The results showed that *Fmo3*, *Cyp17a1*, *Crat*, *Slc22a7*, *Pfkfb3*, *Gstp1*, and *Cyp7b1* may be core genes affecting lipid metabolism and inflammation in liver function (Figure 3C). Consistent with this, the *Fmo3*, *Cyp17a1*, and *Crat* genes were all significantly overexpressed in liver tissue, particularly *Fmo3* (Figure 3D). This suggests that FMO3 may play a significant role in the hepatic metabolic dysfunction and inflammation in silicon-exposed mice.

### 3.4. CS-Activated Macrophages Promote the Expression of Hepatocyte FMO3 In Vitro

**Figure 4:**
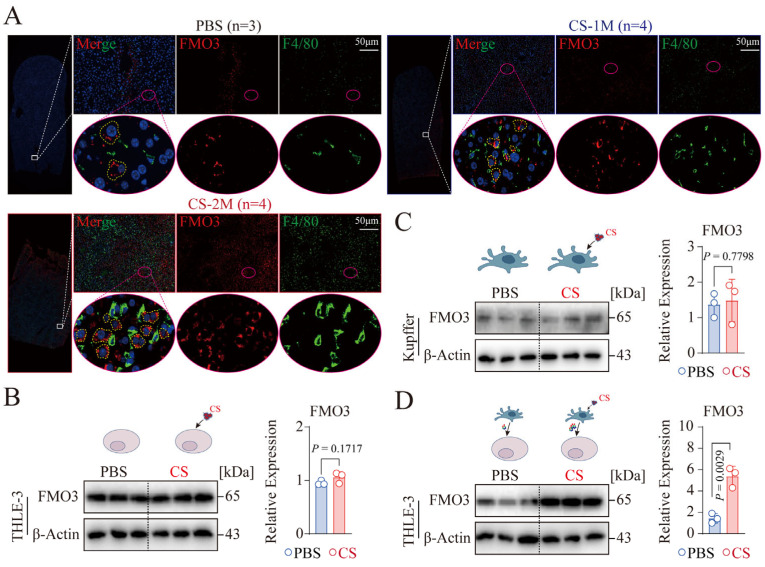
Analysis of FMO3 localization and expression in liver tissue. (**A**). Immunofluorescent staining for FMO3 and F4/80 in the liver tissues of mice with PBS (*n* = 3), CS-1M (*n* = 4), and CS-2M (*n* = 4). The upper inset on the right is a magnified view of the area with in the white solid-line box, the lower inset is a magnified view of the area within the red dashed-line box, and the yellow dashed-line box highlights the area where FMO3 is expressed. (**B**,**C**). Expression levels of FMO3 protein in THLE-3 (**B**) and Kupffer cells (**C**). Following 48 h of stimulation with CS (50 μg/cm^2^), FMO3 protein expression was assessed by western blot analysis, and the experiment was conducted in triplicate. (**D**). Expression levels of FMO3 protein in THLE-3 cells. Following a 48 h stimulation of Kupffer cells with CS (50 μg/cm^2^), the supernatant was harvested and mixed with complete medium at an equal ratio to create conditioned medium (CM). After incubating THLE-3 cells with CM for 48 h, FMO3 protein expression was measured by western blot, with the experiment being performed in triplicate. Scale bar, 50 µm (**A**). Data are displayed as the mean ± SEM, with *p*-values ascertained through independent *t*-tests (**B**–**D**).

Flavin-containing monooxygenase 3 (FMO3) is a microsomal enzyme expressed in the liver, involved in the oxidative metabolism of various drugs and xenobiotics in the body [28]. Previous studies have reported that high expression of FMO3 in liver tissue can promote VLDL levels and secretion in plasma and inhibit the expression of liver X receptor (LXR) and cholesterol synthesis-related genes, leading to metabolic disorders [29]. Recent research has found that the FMO3 product TMAO induces a deficiency in the endoplasmic reticulum calcium recycling channel Serca2 by increasing NLRP3 inflammasome-related cytokines, leading to islet β-cell dysfunction [30]. Similarly, we examined the expression levels of FMO3 and key macrophage (F4/80) cells that mediate inflammation in liver tissue [31]. The results showed that FMO3 was highly expressed in hepatic parenchymal cells (indicated by yellow dashed lines), not in macrophage cells (Figure 4A). Concurrently, compared to the PBS group, the expression of FMO3 (in red) and F4/80 (in green) increased progressively in the CS-1M and CS-2M groups (Figure 4A). This indicates that CS may promote the expression of FMO3 in hepatic parenchymal cells and the infiltration of macrophage cells. Consequently, we stimulated THLE-3 (normal human hepatocytes) and human liver Kupffer cells (liver macrophages) with CS (50 μg/cm^2^) for 48 h and then assessed FMO3 expression. The results showed that compared to Kupffer cells, FMO3 was significantly highly expressed in THLE-3 cells (Figure 4B,C), consistent with the expression of FMO3 in the liver tissue of silicon-exposed mice. Surprisingly, CS stimulation did not promote the expression of FMO3 in THLE-3 and Kupffer cells (Figure 4B,C). To date, no studies have reported the impact of CS on the expression of FMO3 in hepatic parenchymal and Kupffer cells; however, the latest research indicates that Kupffer cells exacerbate the inflammatory state of the liver and impair liver function by releasing inflammatory factors [32]. Based on this, we treated THLE-3 cells with the supernatant after CS-stimulated activation of Kupffer cells and found a significant overexpression of FMO3 (Figure 4B,D). This indicates that activated Kupffer cells may secrete cytokines that promote the expression of FMO3 in THLE-3.

### 3.5. CS-Activated Macrophages Secrete a Large Amount of IL-6

**Figure 5:**
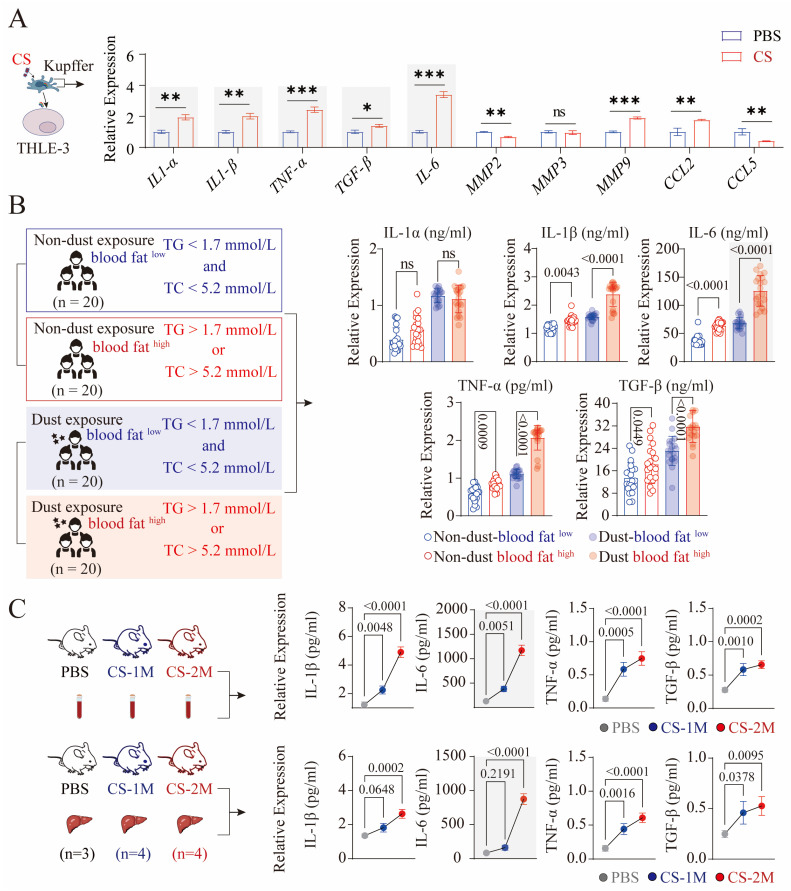
Expression analysis of inflammatory-related factors in clinical samples and mouse tissues. (**A**) Expression levels of inflammatory-related genes in Kupffer cells. After 48 h of stimulation with CS (50 μg/cm^2^), qPCR was used to detect the expression levels of classical inflammatory factors (IL-1α, IL-1β, IL-6, TNF-α, and TGF-β), MMPs (MMP2, MMP3, and MMP9), and CCLs (CCL2 and CCL5), with the experiment repeated three times. (**B**) ELISA was used to measure the expression levels of IL-1α, IL-1β, IL-6, TNF-α, and TGF-β in the serum of dust-exposed (20 cases) and non-dust-exposed individuals (20 cases) with normal and abnormal blood lipid levels. The right side shows a statistical graph of the expression of inflammatory factors, where each dot represents a sample. (**C**) ELISA was used to detect the expression levels of IL-1β, IL-6, TNF-α, and TGF-β in the serum and liver tissues of mice in the PBS (*n* = 3), CS-1M (*n* = 4), and CS-2M (*n* = 4) groups. Data are presented as mean ± SEM, and *p*-values and significance are determined by one-way ANOVA for (**B**,**C**) and two-way ANOVA for (**A**). ns, not significant; * *p* ≤ 0.05; ** *p* ≤ 0.01; *** *p* ≤ 0.001.

Due to the secretion of a large number of inflammatory factors by activated Kupffer cells leading to liver damage [28], we detected the expression of relevant inflammatory factors in CS-activated Kupffer cells, and the results showed that classical inflammatory factors *(IL-1α*, *IL-1β*, *IL-6*, *TNF-α*, and *TGF-β*), MMPs (*MMP9*), and CCLs (*CCL5*) were all highly expressed, and the expression of IL-6 was the most significant (Figure 5A).

Considering the heterogeneity of in vivo and in vitro environments, we randomly selected 20 blood samples each from dust-exposed and non-dust-exposed individuals with normal and abnormal blood lipid levels. We measured the expression levels of inflammatory factors (IL-1α, IL-1β, IL-6, TNF-α, and TGF-β) in the serum. The results showed that in the normal blood lipid group, the levels of IL-1β, IL-6, TNF-α, and TGF-β in the serum of dust-exposed individuals were all upregulated to varying degrees, with the most pronounced trend observed in TGF-β (Figure 5B). In the serum of the abnormal blood lipid group, IL-1β, IL-6, TNF-α, and TGF-β were all significantly upregulated, and the upregulation trend was significantly higher than that of the dust-exposed individuals with normal blood lipids, with IL-1β, IL-6, and TNF-α being the most evident (Figure 5B). Correlation analysis revealed a positive association between the expression of IL-6 and inflammatory factors (IL-1α, IL-1β, TNF-α, and TGF-β) in the blood of all four groups. Among them, the correlation between IL-6 and IL-1β expression was the strongest (Supplementary Figure S2A)

Consistently, the expression of relevant inflammatory factors in the serum and liver tissue of silicon-exposed mice was examined. The results showed that compared with the PBS group, the expression of IL-1β, IL-6, and TNF-α in the serum of silicon-exposed mice was upregulated, with the most significant expression of IL-6 and TNF-α in the CS-2M group (Figure 5C). Correlation analysis revealed a significant positive correlation between IL-6 and IL-1β in the blood of three groups of mice (Supplementary Figure S2B). Similarly, the expression of IL-6 and TNF-α in the liver tissue of mice was upregulated, but only the change in IL-6 in the CS-2M group was the most significant (Figure 5C). Correlation analysis demonstrated a significant positive association between IL-6 and IL-1β in the liver tissue of the three groups of mice (Supplementary Figure S2C). Considering that severe pathological changes only occur in the liver tissue of silicon-exposed mice in the CS-2M group, based on the above results, we speculate that the high expression of IL-6 in the liver tissue of silicon-exposed mice is a key inflammatory factor affecting liver function.

### 3.6. Activation of Macrophages Facilitates Hepatocyte FMO3 Expression via the IL-6/PKC/YY1 Signaling Pathway

**Figure 6:**
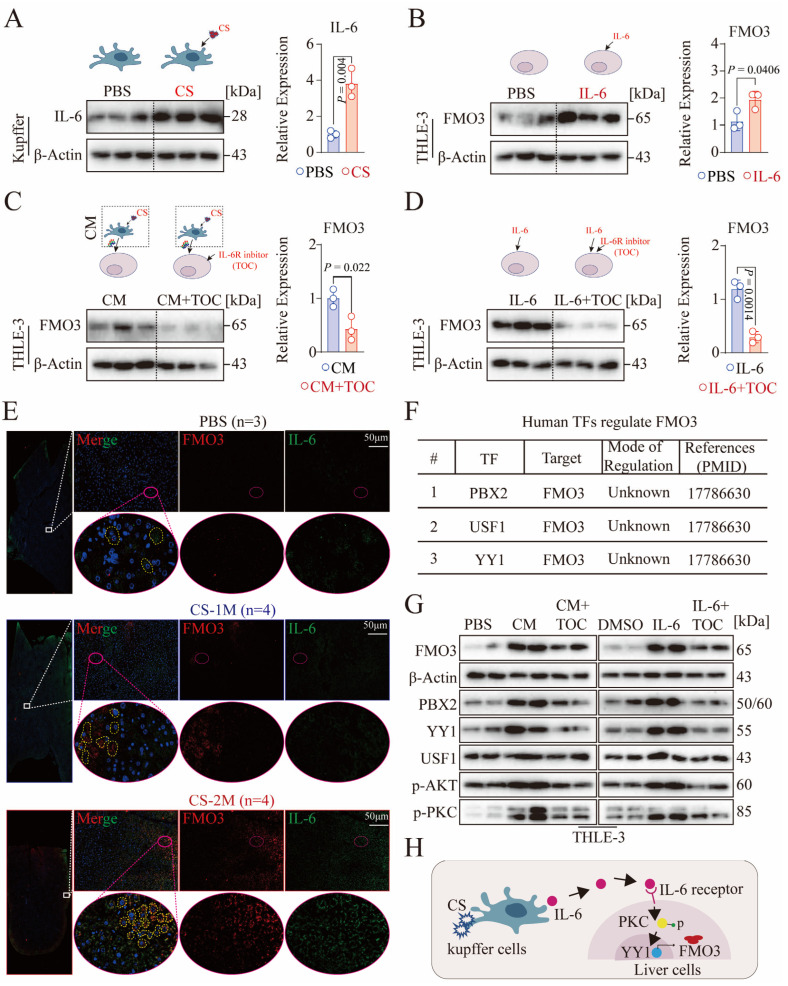
Analysis of the mechanism by which activated macrophages promote the expression of hepatic FMO3. (**A**) IL-6 protein expression levels in Kupffer cells. After 48 h of stimulation with CS (50 μg/cm^2^), IL-6 protein expression was detected by western blot, with the experiment repeated three times. (**B**) FMO3 protein expression levels in THLE-3 cells. After 48 h of stimulation with recombinant IL-6 protein (5 ng/mL), FMO3 protein expression was detected by western blot, with the experiment repeated three times. (**C**) FMO3 protein expression levels in THLE-3 cells under conditioned medium (CM) stimulation, with IL-6R inhibitor (TOC) at 10 ng/mL added for 48 h, followed by western blot detection of FMO3 protein expression, with the experiment repeated three times. (**D**) FMO3 protein expression levels in THLE-3 cells under stimulation with recombinant IL-6 protein and with IL-6R inhibitor (TOC) at 10 ng/mL for 48 h, followed by western blot detection of FMO3 protein expression, with the experiment repeated three times. (**E**) Immunofluorescence staining of FMO3 and IL-6 in liver tissues of mice treated with PBS (*n* = 3), CS-1M (*n* = 4), and CS-2M (*n* = 4). The upper inset on the right shows a high-magnification image of the area enclosed by the white solid line frame, and the lower inset shows a high-magnification image of the area enclosed by the red dashed line frame, with the yellow dashed line frame indicating the FMO3 expression area. (**F**) Identification of key transcription factors regulating the FMO3 gene using the TRRUST database. (**G**) Detection of key kinases (AKT, PKC) and transcription factors (PBX2, YY1, and USF1) involved in IL-6 regulation of FMO3 expression, with the experiment repeated three times. (**H**) Schematic diagram of the pathway by which CS promotes high expression of hepatic FMO3. Scale bar, 50 micrometers (**E**) Data are presented as mean ± SEM, and *p*-values are determined by independent sample *t*-tests (**A**–**D**).

IL-6 (interleukin-6), as a crucial inflammatory cytokine, plays a multifaceted role in liver metabolism and immune regulation [30]. Recent studies report that Kupffer cells can secrete IL-6 to promote the dedifferentiation and regeneration of hepatocytes [33]. Similarly, we found a significant overexpression of IL-6 in CS-activated Kupffer cells (Figure 6A). Additionally, using IL-6 recombinant protein (5 ng/mL) to stimulate THLE-3 cells for 48 h resulted in a significant upregulation of FMO3 (Figure 6B). This indicates that IL-6 secreted by Kupffer cells can promote high expression of FMO3 in hepatocytes.

To differentiate the efficiency of other cytokines secreted by Kupffer cells and IL-6 on the expression of hepatocyte FMO3, we used an IL-6R inhibitor (tocilizumab) at 10 ng/mL under conditions containing CM and, after treating THLE-3 cells for 48 h, found a significant reduction in FMO3 expression (Figure 6C). Consistently, under IL-6 stimulation, the use of an IL-6R inhibitor can also suppress the expression of FMO3 in THLE-3 cells (Figure 6D). This suggests that IL-6 may be a key inflammatory factor in inducing the expression of hepatocyte FMO3. Consistent with this, immunofluorescence was used to detect the expression of FMO3 and IL-6 in liver tissue. The results showed that compared to the PBS group, the expression of both FMO3 and IL-6 significantly increased in the CS-1M and CS-2M groups (Figure 6E and Figure S3A). Correlation analysis revealed a significant positive association between IL-6 and FMO3 expression (Supplementary Figure S3B), suggesting that IL-6 may be a key factor regulating FMO3 expression.

Furthermore, the TRRUST database search revealed that PBX2, USF1, and YY1 are key transcription factors regulating the expression of the FMO3 gene (Figure 6F). Moreover, in vitro cellular experiments discovered that IL-6 can regulate YY1 to affect the expression of FMO3 (Figure 6G and Figure S3C,D). IL-6 can activate various kinases, such as AKT, PKA, and PKC, and YY1 can be activated by both AKT and PKC [34]. Consistent with this, our detection found that IL-6 can promote the activation of PKC kinase in hepatocytes (Figure 6G and Figure S3C,D). This suggests that IL-6 may regulate the expression of FMO3 in hepatocytes through the PKC/YY1 signaling pathway, affecting liver metabolism (Figure 6H).

## 4. Discussion

Exposure to coal (silica) dust is a primary health hazard for coal miners. Long-term exposure not only increases the risk of pneumoconiosis but is also associated with various chronic diseases such as hypertension, diabetes, dyslipidemia, and hyperuricemia [35,36].

Our study found that among 5433 samples, the proportion of dyslipidemia in the dust-exposed group was as high as 65.7%, significantly higher than the 21.6% in the non-dust-exposed group. This reveals a strong correlation between dust exposure and dyslipidemia. Dyslipidemia poses a serious threat to health, mainly focusing on pathophysiology and the risk of cardiovascular diseases [37,38]. However, the correlation and mechanism between coal (silica) dust exposure and dyslipidemia are not yet clear. Rajagopalan and colleagues’ research indicates that particulate matter, especially PM2.5, is a major threat to the increased cardiovascular mortality rate [39]. Consistently, PM2.5 can also accelerate atherosclerosis through oxidative stress, inflammation, and abnormal accumulation of oxidized lipids in the vascular system [40,41]. Furthermore, Li et al. found that PM2.5 exposure leads to changes in stress hormone and metabolite levels, affecting glucose, amino acids, fatty acids, lipids, etc. [42].

This suggests that particulate matter exposure may increase the risk of liver metabolic abnormalities. This study, through the construction of a liver abnormality animal model exposed to silica, found that after 30 days of silica exposure, inflammatory nodules were produced in the lungs, but only a small amount of silica accumulated in the liver without obvious pathological changes. After 60 days, a large accumulation of silica in the liver was accompanied by fatty-like lesions. At the same time, the mice also exhibited abnormal liver inflammatory responses and blood lipid levels. The liver is a key organ for regulating lipid metabolism, and hepatocytes are the main regulatory cells for lipid metabolism [43,44,45]. Tissue sequencing and sample fluorescence detection identified flavin-containing monooxygenase 3 (FMO3) as an abnormally overexpressed gene in the livers of silica-exposed mice, and the overexpression is in hepatocytes, not other cells. FMO3, as a liver microsomal enzyme, is involved in the metabolism of various substances [46]. Studies have found that the high expression of FMO3 is associated with an increase in plasma VLDL levels and the inhibition of cholesterol synthesis, which may lead to metabolic disorders [47]. Its metabolic product TMAO is associated with cardiovascular diseases, and the latest research shows that TMAO may affect the function of pancreatic β-cells through the NLRP3 inflammasome [30]. This suggests that FMO3 may play an important role in the metabolic abnormalities and inflammation of liver function in silica-exposed mice.

The inflammatory response of liver tissue is mediated by various inflammatory factors, among which Kupffer cells play a central role [48,49]. We found that silica particles can induce the activation of liver Kupffer cells to secrete inflammatory factors such as interleukin-6 (IL-6), activating the immune response in the liver microenvironment. Clinical and animal sample tests found that the levels of IL-6 in the serum of dust-exposed workers and mice, as well as in liver tissue, were significantly elevated in the dyslipidemia group. IL-6 can promote fat synthesis and is associated with elevated IL-6 levels in the serum of obese children [50]. Furthermore, IL-6 in liver homogenates is closely associated with the degree of liver steatosis and may be a significant cytokine in non-alcoholic fatty liver disease (NAFLD). Finally, our findings suggest that IL-6 might modulate the expression of FMO3 within hepatocytes via the PKC/YY1 signaling cascade, which in turn could impact hepatic metabolism and lipid dysregulation.

In summary, this research highlights the significance of addressing cardiovascular chronic diseases among individuals exposed to coal dust, offering scientific evidence for the enhancement of health monitoring protocols for such exposures. Although potential limitations exist due to discrepancies between the murine model and real-world scenarios, this research offers significant information on the effects of coal dust on liver functionality and sets a course for upcoming studies.

## Data Availability

All data supporting the findings of this study are available within the paper and its Supplementary Information, with the sequencing data for mouse liver tissue provided in Supplementary Table S1.

## Supplementary Materials

The following supporting information can be downloaded at https://www.mdpi.com/article/10.3390/genes16040456/s1: Table S1: Transcriptome sequencing data of liver tissue; Table S2: Lists of primers; Table S3: Metabolism and inflammation pathway-related genes. Figure S1. CONSORT flow-diagram. Figure S2. Correlation analysis between IL-6 and other inflammatory cytokines ex-pression. A. Correlation analysis between the expression of IL-6 and inflammatory cytokines (IL-1α, IL-1β, TNF-α, and TGF-β) in the blood of four groups of individuals. B, C. Correlation analysis between IL-6 expression and inflammatory cytokines (IL-1α, IL-1β, TNF-α, and TGF-β) in the blood (B) and liver tissue (C) of three groups of mice. The Pearson Correlation Coefficient (R) indicates the correlation, and *p* < 0.05 is considered statistically significant. Figure S3. Statistics of FMO3, IL-6, and related protein expression in liver tissue and cells. (A). Statistical analysis of fluorescence intensity for FMO3 and IL-6 in liver tissue from three groups of mice. (B). Correlation analysis of fluorescence intensity for FMO3 and IL-6 in liver tissue from three groups of mice. (C). Bar chart showing the expression statistics of FMO3 and related pathway pro-teins in THLE-3 cells under PBS, CM, and CM+TOC treatment conditions. (D). Bar chart demon-strating the expression statistics of FMO3 and related pathway proteins in THLE-3 cells under PBS, IL-6, and IL-6+TOC treatment conditions. Data are presented as Mean ± SEM, and P values and significance are determined by One-Way ANOVA for (A,C,D).
