# Supplementary figures and images for: IL-6 Affects Liver Metabolic Abnormalities Caused by Silicon Exposure by Regulating the PKC/YY1 Signaling Pathway
Source: Genes (Basel). 2025 Apr 16;16(4):456. doi: 10.3390/genes16040456 (PMC12026785; doi:10.3390/genes16040456)

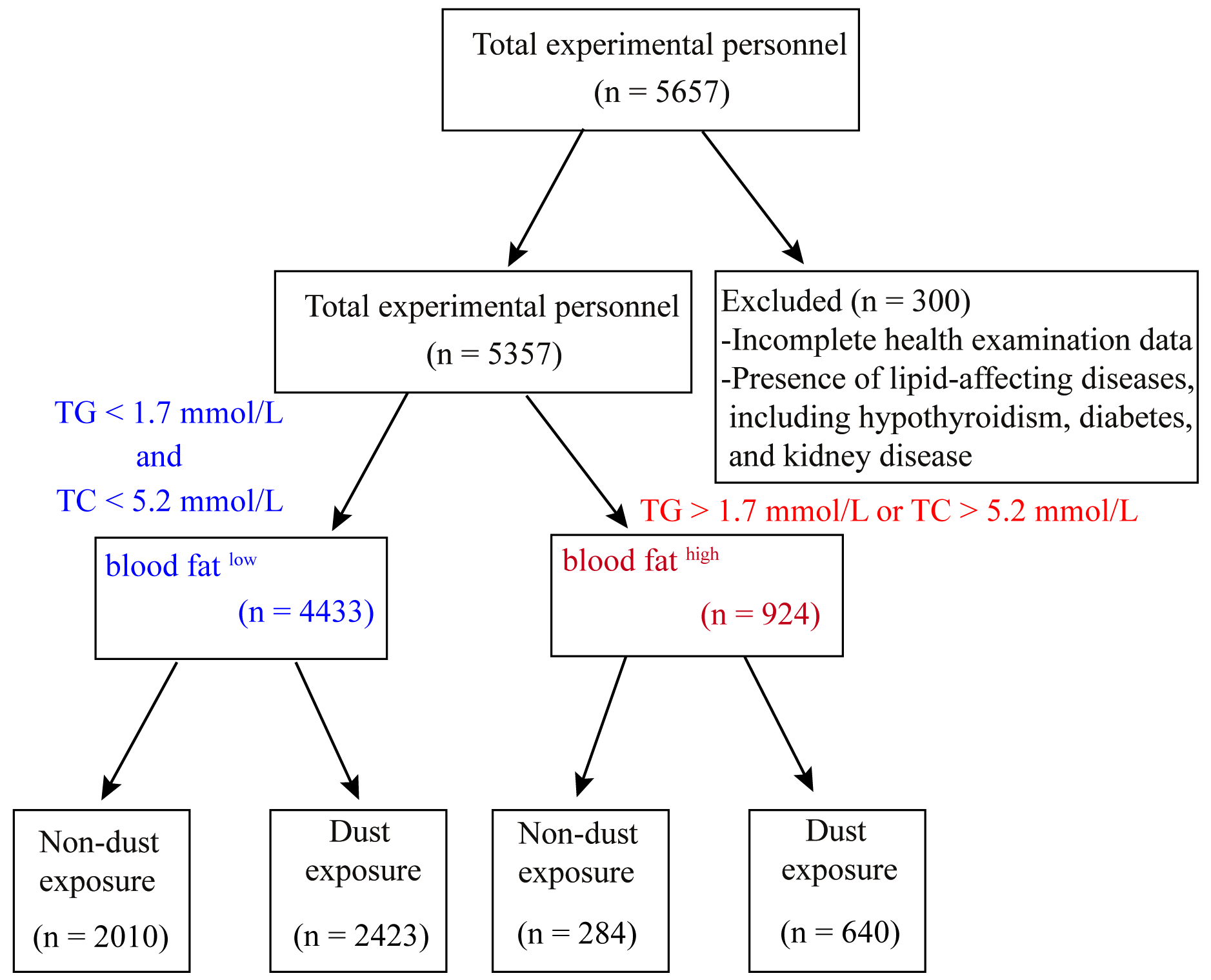

Supplement: Supplementary file 1 [file genes-16-00456-s001.zip › Supplementary Fig.S1.tif]

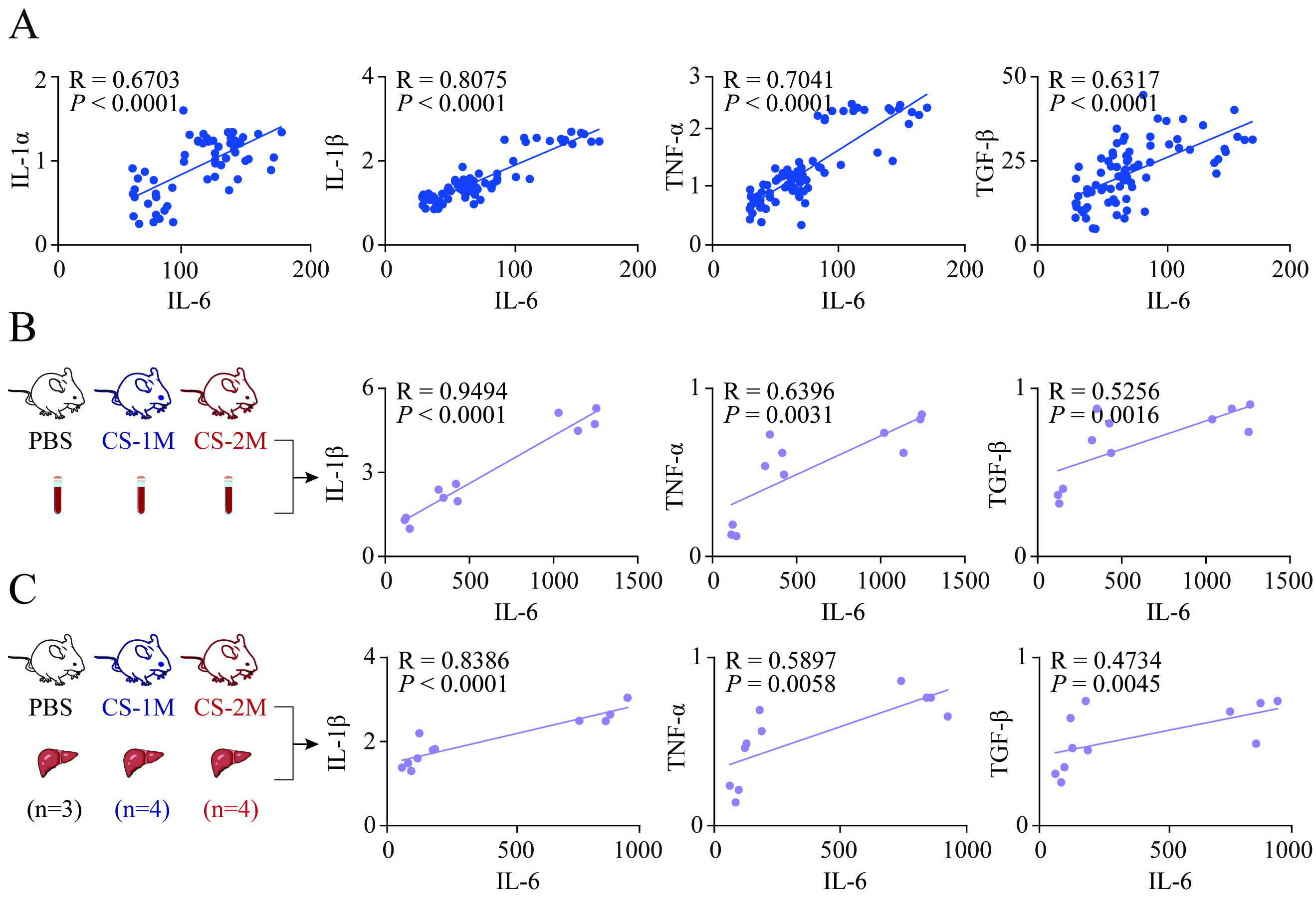

Supplement: Supplementary file 1 [file genes-16-00456-s001.zip › Supplementary Fig.S2.tif]

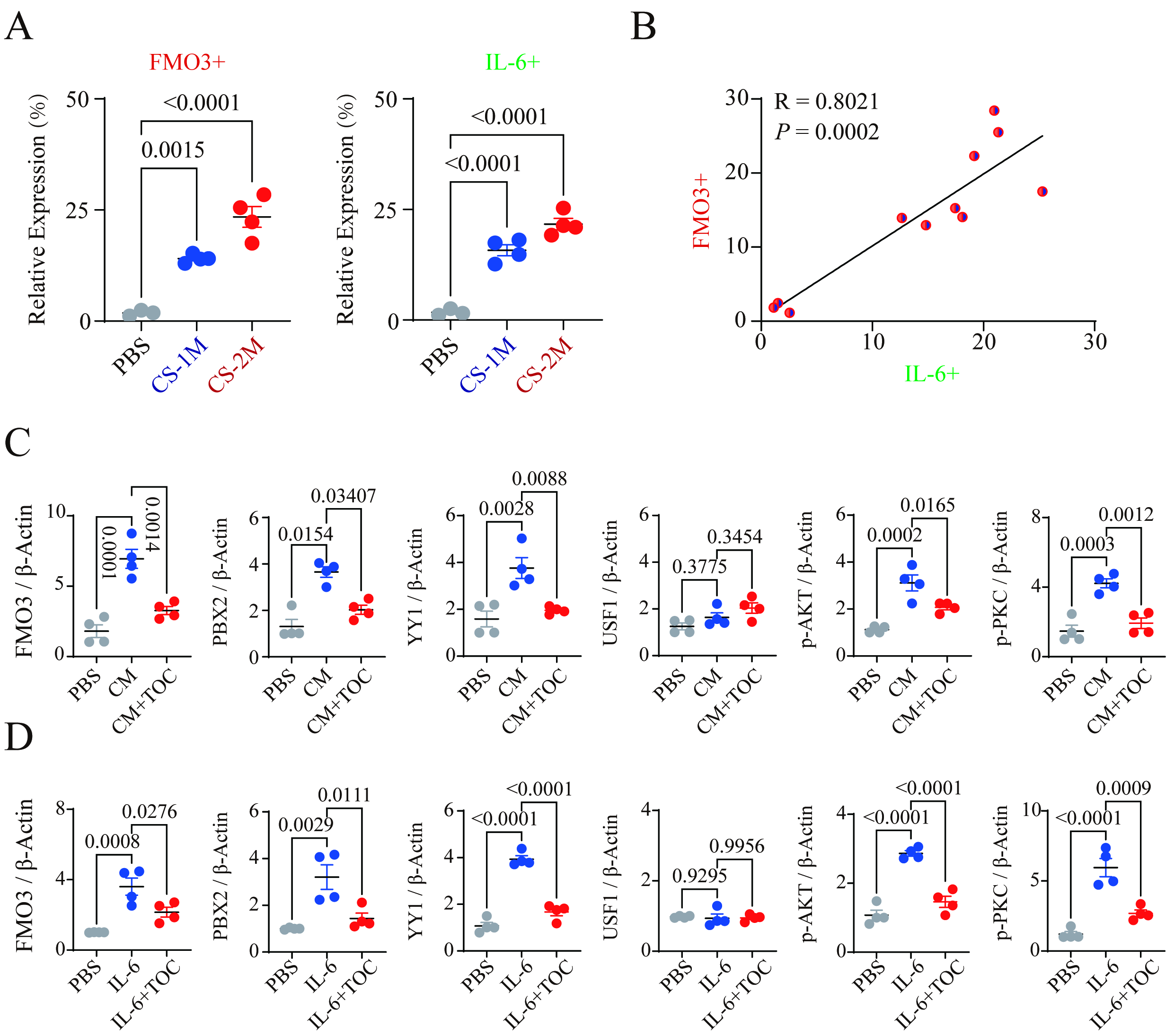

Supplement: Supplementary file 1 [file genes-16-00456-s001.zip › Supplementary Fig.S3.tif]
